# Supplementary material for: Targeted delivery of the probiotic Saccharomyces boulardii to the extracellular matrix enhances gut residence time and recovery in murine colitis
Source: Nat Commun. 2024 May 6;15:3784. doi: 10.1038/s41467-024-48128-0 (PMC11074276; doi:10.1038/s41467-024-48128-0)
Supplement: Supplementary file 1 — Supplementary Information [file 41467_2024_48128_MOESM1_ESM.pdf]

Supplementary Information

Targeted delivery of the probiotic *Saccharomyces boulardii* to the extracellular matrix enhances gut residence time and recovery in murine colitis

Mairead K. Heavey[1], Anthony Hazelton[1], Yuyan Wang[1], Mitzy Garner[1], Aaron Anselmo[1†], Janelle C. Arthur[2,3,4*], Juliane Nguyen[1,4*]

[1]Division of Pharmacoengineering and Molecular Pharmaceutics, Eshelman School of Pharmacy, University of North Carolina at Chapel Hill, Chapel Hill, NC 27599, USA

[2] Department of Microbiology and Immunology, The University of North Carolina at Chapel Hill, Chapel Hill, NC, United States

[3] Center for Gastrointestinal Biology and Disease, The University of North Carolina at Chapel Hill, Chapel Hill, NC, United States

[4] Lineberger Comprehensive Cancer Center, The University of North Carolina at Chapel Hill, Chapel Hill, NC, United States

[†]Present address: VitaKey Incorporation, Durham, NC 27701

***Correspondence to:**

Juliane Nguyen, email: julianen@email.unc.edu

Janelle C. Arthur, email: janelle_arthur@med.unc.edu

This file includes:

Supplementary Figures 1-6

Supplementary Table 1

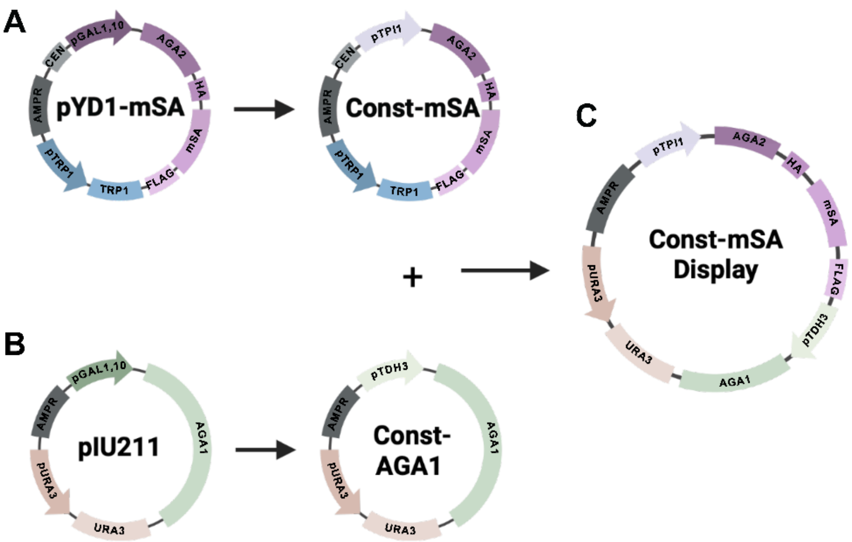

Supplementary Figure 1. Plasmid generation of stable mSA surface expression in *S.b.* Schematic depicting steps involved in the generation of a single surface display plasmid to enable genomic integration and constitutive expression of mSA on the surface. (**A-B**) Galactose inducible promoters (pGAL1,10) in commercially available yeast-surface-display plasmids, pYD1-mSA and pIU211, were replaced with constitutive yeast promoters, pTPI1 and pTDH3, respectively. (**C**) Essential components from Const-mSA and Const-AGA1 plasmids, (AGA1 expression cassette, AGA2-mSA expression cassette, bacterial ampicillin resistance selection marker, uracil auxotrophic marker selection cassette) were combined to generate a single plasmid amenable to stable genomic integration. Plasmid sequence can be found in **Supplementary Table 1** (below).

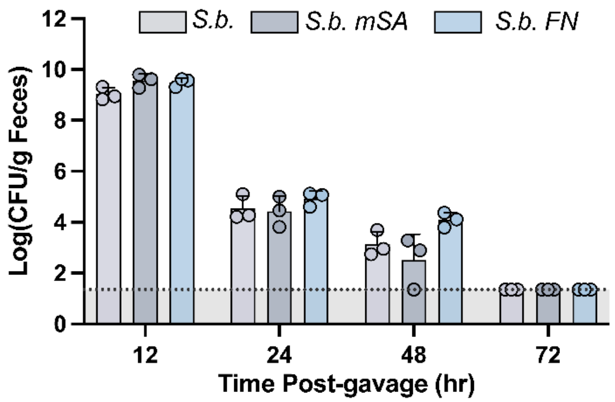

Supplementary Figure 2. *S. boulardii* pharmacokinetics in healthy mice. Healthy, 6- to 8-week-old, female C57BL/6J mice were orally gavaged with 10^9 *S.b.*, *S.b. mSA*, or *S.b. FN*. Fresh fecal samples were collected at the timepoints indicated to measure yeast concentrations. Data are represented as mean ± SD, n = 3 independent animals. Dotted line represents the limit of detection.

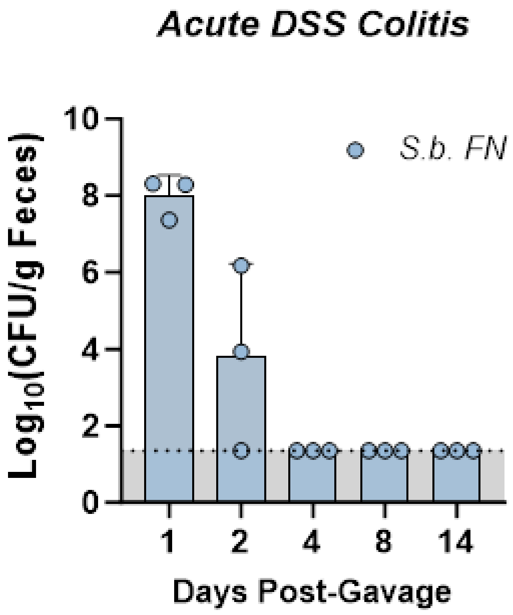

Supplementary Figure 3. Long-term *S.b.* pharmacokinetic profiles in an acute model of DSS of colitis. Fecal CFU following administration of 10^9 *of S.b. FN* in an acute model of DSS colitis. Data are represented as mean ± SD, n = 3 (independent animals), dotted line represents the limit of detection.

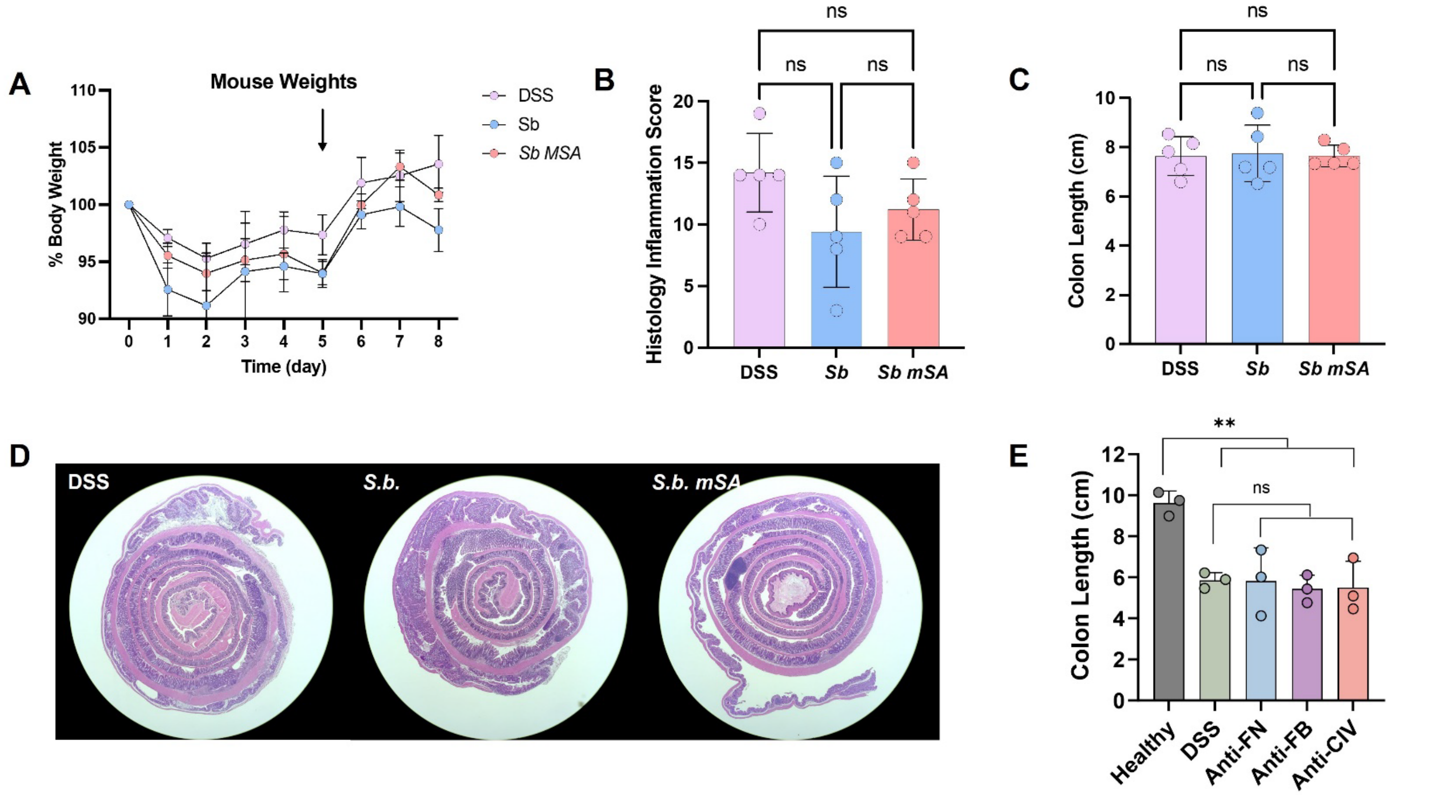

Supplementary Figure 4. (A, B, C, D) Sb and Sb mSA show similar effects with no statistical differences in in acute DSS colitis. (E) ECM-targeting antibodies alone exert no significant effect. (A) Mean percent body weight of mice over the course of the study, n=5, significance indicates comparisons to average weights of DSS-only mice at each timepoint. Arrow indicates day of DSS removal and yeast dosing. **(B)** Semi-quantitative histological scores of inflammation accounting for

extent of mucosal loss, hyperplasia, and erosions, n = 5 independent animals. **(C)** Colon lengths following administration of engineered S. boulardii in acute DSS colitis model, n = 5 independent animals. **(D)** Representative images of hematoxylin and eosin staining of colon Swiss rolls. (E) Colon lengths following administration ECM-targeting antibodies in acute DSS colitis model, n = 5 independent animals. The acute DSS model was established as described under the Methods section. When switched back to normal drinking water, mice were orally gavaged with 30 µg biotin-anti-fibronectin (Anti-FN), biotin-anti-fibrinogen (Anti-FB), or biotin-anti-collagen IV (Anti-CIV) (Abcam, ab6584, ab51416, or ab6581) antibodies resuspended in 150 µL sterile PBS. The 30 µg antibody dose was chosen as this is the equivalent dose of antibody after incubation with *S.b. mSA* for targeted *S.b.* administration in Figures 4 and 5. Data are represented as mean ± SD. Significance was assessed using ordinary one-way ANOVA with Tukey's multiple comparisons test α = ** $p < 0.01$.

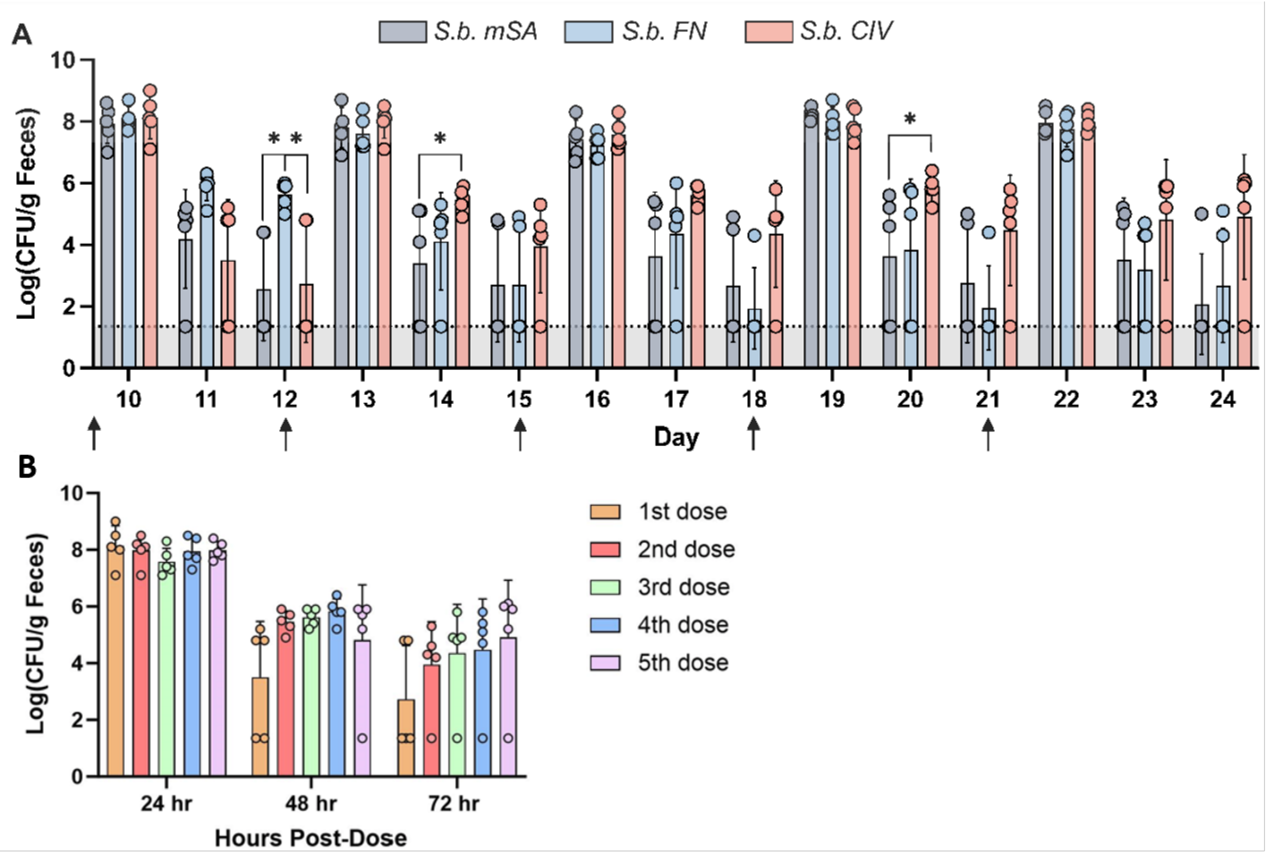

Supplementary Figure 5. Fecal CFU of *S.b.* over time in a relapsing-remitting model of DSS colitis. (**A**) Fecal yeast concentrations at all timepoints indicated in Fig. 5 (n = 5 independent animals). Bars represent mean, error shown as standard deviation, significance assessed using ordinary one-way ANOVA with Kruskal-Wallis multiple comparisons test α = 0.05, * $p < 0.05$. (**B**) In-depth analysis of fecal CFU over time in the *S.b. CIV* treated group at timepoints 24 hours, 48 hours, and 72 hours post-dose. Significance was assessed using ordinary one-way ANOVA with Tukey's multiple comparisons test. No significant differences were found between the groups.

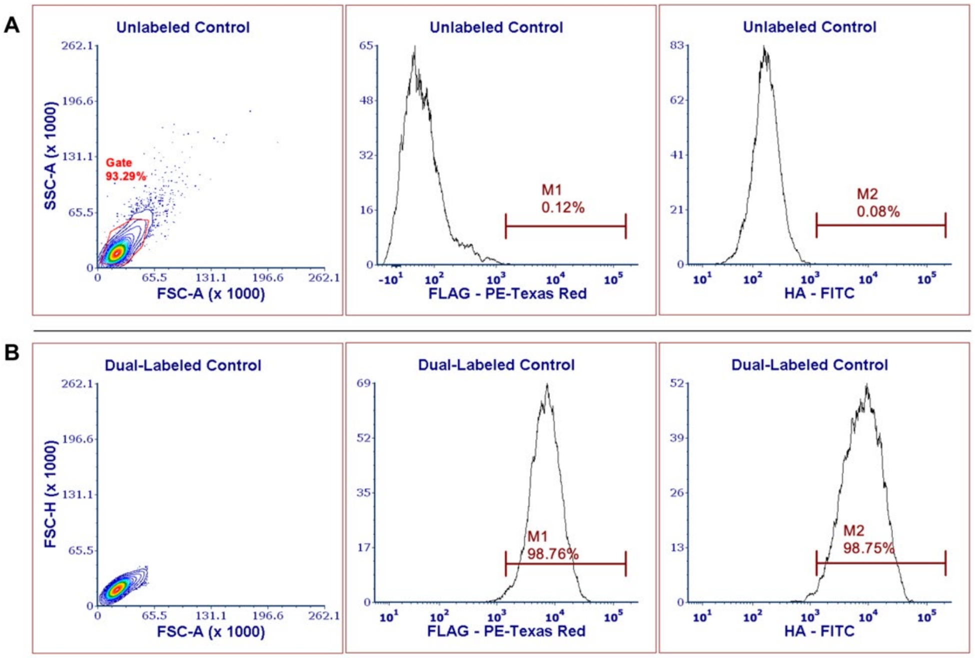

C

Filename	Parameter	Overlay Gate	# of Events	% of gated cells (from plot)	Median	Geometric Mean	CV
Controls_HA_FLAG_005.fcs	FITC-A	Gate	9217	98.75	7783.08	7581.43	72.48
Controls_Unlabeled_001.fcs	FITC-A	Gate	7	0.08	1491.52	1695.92	32.40

Filename	Parameter	Overlay Gate	# of Events	% of gated cells (from plot)	Median	Geometric Mean	CV
Controls_HA_FLAG_005.fc	PE-Texas	Gate	9254	99.14	6719.44	6654.60	59.86
Controls_Unlabeled_001.fc	PE-Texas	Gate	12	0.13	1712.10	1910.38	43.70

Supplementary Figure 6. Representative plots of flow cytometry gating strategy. (**A**) Contour plot and histogram plots (PE-Texas-Red and FITC) for negative control, unlabeled yeast cells. 'Gate' in the contour plot indicates yeast cell gating strategy. M1 and M2 indicate marker gating strategy to identify positive fluorescently labeled cells. (**B**) Contour plot and histograms resulting from the positive control of dual-labeled yeast cells (FLAG-PE-TexasRed and HA-FITC). (**C**) Histogram statistics for all histogram plots. Gating strategies are representative for all flow cytometry experiments (Figs. 1e and 2e).

Supplementary Table 1 – Plasmid sequence of Supplementary Figure 1

ScTDH3 promoter	Cagttcgagtttatcattatcaatactgccatttcaaagaatacgtaaataattaatagtagtgattttcctaactttatttagtcaaaaaattagccttttt<br>aattctgctgtaacccgtacatgcccaaaatagggggcgggttacacagaatatataacatcgtaggtgtctgggtgaacagtttattcctggcat<br>ccactaaatataatggagcccgctttttaagctggcatccagaaaaaaaaagaatcccagcaccaaaatattgttttcttcaccaaccatcagtt<br>cataggtccattctcttagcgcaactacagagaacaggggcacaaacaggcaaaaaacgggcacaacctcaatggagtgatgcaacctgc<br>ctggagtaaatgatgacacaaggcaattgacccacgcatgtatctatctcattttcttacaccttctattaccttctgctctctctgatttggaaaaagc<br>tgaaaaaaaaggttgaaaccagttccctgaaattattcccctacttgactaataagtatataaagacggtaggtattgattgtaattctgtaaatcta<br>tttcttaaacttcttaaattctacttttatagttagtcttttttttagttttaaaacaccaagaacttagtttcgaataaacacacataaacaaacaaa
Aga1	atgacattatctttcgctcattttacctacctgttcacaatattgttgggattaactaatattgccttggcatctgatccagaaacgattctagtgacgat<br>aaccaagacaaacgatgcaaatggggttgttacaactacagtttcacccgcgctagtctccacatccactatcgttcaagctggcactacgaca<br>ttgtatacgacttggtgtccattgacggtatccacttcatctgctgccgaaataagtccttcaatatcgtacgctactaccctatccagatttagtacttt<br>gacattatctacagaagtctgctcccatgaggcatgtccttcgtcatcgacgttgccaaccaccaccttatctgtgacttccaagttcacttcatatat<br>ttgccctacttgtcacacaaccgctatcagctcattatccgaagtaggaactacaaccgtggtatcatccagcgccattgaaccatcaagtgcct<br>ctataatctcacctgtcacctctacactttcgagtacaacatcgtccaatccaactactacctccctaagttcgacatctacatctccaagctctacat<br>ctacatctccaagctctacatctacctcatcaagttcgacatctacctcatcaagttcgacatctacctcatcaagttcgacatctacatctccaagtt<br>cgacatccacatcttcaagtttgacatccacatcttcaagttctacatctacatcccaaagttctacatctacctcatcaagttcgacatctacatctc<br>caagctctacatctacctcatcaagttcaacatctacatctccaagttctaaatctacttctgcaagctccacttccacttcttcatattcaacatctac<br>atccccaagtttgacttcttcatctccaactttggcttccacttctccaagttcaacatctattagctctacttttactgattcaacttcatcccttggctcct<br>ctatagcatcttcatcaacgtctgtgtcattatacagcccatccacacctgtttactccgtcccttcgacttcgtcaaatgttgcaactccttctatgactt

	cttcaactgttgaaacaactgttagttcacaaagttcgtctgaatatatcaccaaatcctcaatttctactactatcccatcattttccatgtctacatatttcaccactgttagtggagtcactacaatgtatacgacatggtgtccttatagctctgaatctgagactagcacattaaccagtatgcatgaaacggttacaacagacgctacagtctgcactcacgagtcttgcatgccctcgcagacaacaagtttgattacatcttctataaaaatgtccactaaaacgtcgcaacttctgtaagcacctcaacggttgaatcctcatatgcatgctccacatgtgctgaaacgtcacactcgtattcttccgtgcaaacagcttcatcaagttctgtaacacagcagaccacatccacaaagagttgggtaagttcaatgacaacttcggatgaagatttcaataagcacgctaccggtaagtatcatgtaacatcttcaggtacctcaaccatttcgactagtgtaagtgaagccacgagtacatcaagcattgactcagaatctcaagaacaatcatcacacttattatcgacatcggtcctttcatcctcctccttgtctgctacattatcctctgacagtactattttgctattcagttctgtatcatcactaagtgtcgaacagtcaccagttaccacacttcaaatttcttcaacatcagagattttacaacccacttcttccacagctattgctacaatatctgcctctacatcatcactttccgcaacatctatctctacaccatctacctctgtggaatcgactattgaatcttcatcattgactccgacggtatcttctattttcctctcatcatcatctgctccctcttctctacaaacatctgttaccactacagaagtttccactacttcaatctccatacaataccaaacttcatcaatggtaacaattagccaatatatgggcagtggatcgcaaacgcgtttgccattaggaaagttggtcttcgccatcatggcagttgcctgcaatgtaattttcagttaa
tMATa Terminator	tagctcgagatctgataacaacagtgtagatgtaacaaaatcgactttgttcccactgtacttttagctcgtacaaaatacaatatacttttcatttctccgtaaacaacatgttttcccatgtaatatccttttctatttttcgttccgttaccaactttacacatactttatatagctattcacttctatacactaaaaaactaagacaattttaattttgctgcctgccatatttcaatttgttataaattcctataatttatcctattagtagctaaaaaaagatgaatgtgaatcgaatcctaagagaattg
TPI1 promoter	ctaatacattcagacacttctgcggtatcaccctacttattcccttcgagattatatctaggaacccatcaggttggtggaagattacccgttctaagacttttcagcttcctctattgatgttacacctggacacccctttttctggcatccagtttttaatcttcagtggcatgtgagattctccgaaattaattaaagcaatcacacaattctctcggataccacctcggttgaaactgacaggtggtttgttacgcatgctaatgcaaaggagcctatatacctttggctcggctgctgtaacagggaatataaagggcagcataatttaggagtttagtgaacttgcaacatttactattttcccttcttacgtaaatatttttctttttaattctaaatcaatctttttcaatttttttgtttgtattcttttcttgcttaaatctataactacaaaaaacacatacataaactaaaa
Aga2	atgcagttacttcgctgtttttcaatattttctgttattgcttcagttttagcacaggaactgacaactatatgcgagcaaatcccctcaccaactttagaatcgacgccgtactctttgtcaacgactactattttggccaacgggaaggcaatgcaaggagtttttgaatattacaaatcagtaacgtttgtcagtaattgcggttctcacccctcaacaactagcaaaggcagccccataaacacacagtatgtttttaag
HA tag	tacccatacgacgttccagactacgct
Linker	ctgcaggctagtggtggtggtggttctggtggtggtggttctggtggtggtggttctgctagc
mSA	gcggaagcgggtatcaccggcacgtggtacaaccagtctggttctaccttcaccgttaccgcgggtgcggacggtaacctgaccggtcagtacgaaaaccgtgcgcagggcactggttgccagaactctccgtacaccctgaccggtcgttacaacggtaccaaactggaatggcgtgttgaatggaacaactctaccgaaaactgccactctcgtaccgaatggcgtggtcagtaccagggtggtgcggaagcgcgtatcaacacccagtggaacctgacctacgaaggtggttctggtccggcgaccgaacagggtcaggacaccttcaccaaagttaaa
Flag Tag	gactacaaggacgatgacgacaag
