# Supplementary material for: Targeted delivery of the probiotic Saccharomyces boulardii to the extracellular matrix enhances gut residence time and recovery in murine colitis
Source: Nat Commun. 2024 May 6;15:3784. doi: 10.1038/s41467-024-48128-0 (PMC11074276; doi:10.1038/s41467-024-48128-0)
Supplement: Supplementary file 3 — Reporting Summary [file 41467_2024_48128_MOESM3_ESM.pdf]

nature portfolio

Corresponding author(s): Juliane Nguyen and Janelle Arthur

Last updated by author(s): Apr 7, 2024

Reporting Summary

Nature Portfolio wishes to improve the reproducibility of the work that we publish. This form provides structure for consistency and transparency in reporting. For further information on Nature Portfolio policies, see our Editorial Policies and the Editorial Policy Checklist.

Statistics

For all statistical analyses, confirm that the following items are present in the figure legend, table legend, main text, or Methods section.

n/a	Confirmed	
☐	☒	The exact sample size (*n*) for each experimental group/condition, given as a discrete number and unit of measurement
☐	☒	A statement on whether measurements were taken from distinct samples or whether the same sample was measured repeatedly
☐	☒	The statistical test(s) used AND whether they are one- or two-sided<br>*Only common tests should be described solely by name; describe more complex techniques in the Methods section.*
☒	☐	A description of all covariates tested
☐	☒	A description of any assumptions or corrections, such as tests of normality and adjustment for multiple comparisons
☐	☒	A full description of the statistical parameters including central tendency (e.g. means) or other basic estimates (e.g. regression coefficient) AND variation (e.g. standard deviation) or associated estimates of uncertainty (e.g. confidence intervals)
☐	☒	For null hypothesis testing, the test statistic (e.g. *F*, *t*, *r*) with confidence intervals, effect sizes, degrees of freedom and *P* value noted<br>*Give P values as exact values whenever suitable.*
☒	☐	For Bayesian analysis, information on the choice of priors and Markov chain Monte Carlo settings
☒	☐	For hierarchical and complex designs, identification of the appropriate level for tests and full reporting of outcomes
☒	☐	Estimates of effect sizes (e.g. Cohen's *d*, Pearson's *r*), indicating how they were calculated

Our web collection on statistics for biologists contains articles on many of the points above.

Software and code

Policy information about availability of computer code

Data collection	Echo Revolve microscope, BD FACSDiva, Xcalibur, Softmax Pro Molecular Devices, QuantStudio 6 Flex Real-Time PCR System
Data analysis	Microsoft Excel (v.16.7), Prism GraphPad, FCSExpress, Image J, SnapGene

For manuscripts utilizing custom algorithms or software that are central to the research but not yet described in published literature, software must be made available to editors and reviewers. We strongly encourage code deposition in a community repository (e.g. GitHub). See the Nature Portfolio guidelines for submitting code & software for further information.

Data

Policy information about availability of data

All manuscripts must include a data availability statement. This statement should provide the following information, where applicable:

- Accession codes, unique identifiers, or web links for publicly available datasets
- A description of any restrictions on data availability
- For clinical datasets or third party data, please ensure that the statement adheres to our policy

The transcriptional expressions of Fn1, Col4a1, and Fgb from healthy and UC human patients were analyzed from the published GEO datasets (http://www.ncbi.nlm.nih.gov/geo/) obtained from GSE13367, GSE9452, GSE38713, GSE47908, GSE73661, GSE114527, and GSE87466. The data underlying Fig. 1d-f, Fig. 2b, e, f, Fig. 3b-f, Fig. 4b-f,h,i, Fig. 5b-g,i,j, as well as Supplementary Figs. 2-5 are in the associated source data file.

Human research participants

Policy information about studies involving human research participants and Sex and Gender in Research.

Reporting on sex and gender	n/a
Population characteristics	n/a
Recruitment	n/a
Ethics oversight	n/a

Note that full information on the approval of the study protocol must also be provided in the manuscript.

Field-specific reporting

Please select the one below that is the best fit for your research. If you are not sure, read the appropriate sections before making your selection.

[x] Life sciences [ ] Behavioural & social sciences [ ] Ecological, evolutionary & environmental sciences

For a reference copy of the document with all sections, see nature.com/documents/nr-reporting-summary-flat.pdf

Life sciences study design

All studies must disclose on these points even when the disclosure is negative.

Sample size	Sample sizes were estimated based on previous studies (Cao et al. Nat Comms, 2019 and Xu et al. Nat Comms, 2022) and have been listed in the manuscript. The group sizes represent the minimum number of animals per group needed to reach statistical significance ($p < 0.05$).
Data exclusions	No data were excluded.
Replication	All in vitro studies had n=3 replicates. In vivo studies had n=5 mice per group. All n's in these experiments had produced similar results suggesting successful replication and reproducibility. Additionally, pilot studies were conducted for the in vivo studies, and they yielded the same trends and statistically significant results as the repeated full studies.
Randomization	Mice were randomly allocated into experimental groups.
Blinding	Investigators were blinded during sample collection for in vivo studies.

Reporting for specific materials, systems and methods

We require information from authors about some types of materials, experimental systems and methods used in many studies. Here, indicate whether each material, system or method listed is relevant to your study. If you are not sure if a list item applies to your research, read the appropriate section before selecting a response.

Materials & experimental systems

n/a	Involved in the study	
[ ]	[x]	Antibodies
[x]	[ ]	Eukaryotic cell lines
[x]	[ ]	Palaeontology and archaeology
[ ]	[x]	Animals and other organisms
[x]	[ ]	Clinical data
[x]	[ ]	Dual use research of concern

Methods

n/a	Involved in the study	
[x]	[ ]	ChIP-seq
[ ]	[x]	Flow cytometry
[x]	[ ]	MRI-based neuroimaging

Antibodies

Antibodies used	Biotin Anti-Fibronectin (Abcam, ab6584)<br>Biotin Anti-Collagen IV antibody (Abcam, ab6581)<br>Biotin Anti-Fibrinogen antibody (Abcam, ab51416)<br>Anti-Fibronectin antibody (Abcam, ab2413)<br>HA Tag Alexa Fluor® 488-conjugated Antibody (R&D Systems, IC6875G)

DYKDDDDK Epitope Tag Alexa Fluor® 594-conjugated Antibody (R&D Systems, IC8529T)
Goat anti-Rabbit IgG (H+L) Cross-Adsorbed Secondary Antibody, Alexa Fluor™ 594 (Invitrogen, A-11012)
IL-10 Mouse ELISA Kit (Invitrogen, BMS614)
Human Il-8 Cytoset (Invitrogen, CHC1303)
Fibronectin Monoclonal Antibody (NeoBiotechnologies, FN1, 3029)
Rabbit anti-Saccharomyces cerevisiae antibody (BioRad, 8203-0050)
Goat anti-Mouse IgG (H+L) Cross-Adsorbed Secondary Antibody, Alexa Fluor™ 594 (Invitrogen, A-11005)
Goat anti-Rabbit IgG (H+L) Cross-Adsorbed Secondary Antibody, Alexa Fluor™ 647 (Invitrogen, A-21244)

Validation

Validation of all primary and secondary antibodies can be found on the manufacturer’s website.

Animals and other research organisms

Policy information about studies involving animals; ARRIVE guidelines recommended for reporting animal research, and Sex and Gender in Research

Laboratory animals

Six- to eight-week-old female C57BL/6J mice housed in groups of 5 were used for all in vivo studies. Mice were purchased from The Jackson Laboratory. Mice were separated to 2-3 mice per cage and acclimated for at least 72 hours prior to study initiation.

Wild animals

n/a

Reporting on sex

n/a

Field-collected samples

n/a

Ethics oversight

Animal studies were conducted in accordance with and approved by the Institutional Animal Care and Use Committee (IACUC) of The University of North Carolina at Chapel Hill.

Note that full information on the approval of the study protocol must also be provided in the manuscript.

Flow Cytometry

Plots

Confirm that:

- [x] The axis labels state the marker and fluorochrome used (e.g. CD4-FITC).
- [x] The axis scales are clearly visible. Include numbers along axes only for bottom left plot of group (a 'group' is an analysis of identical markers).
- [x] All plots are contour plots with outliers or pseudocolor plots.
- [x] A numerical value for number of cells or percentage (with statistics) is provided.

Methodology

Sample preparation

Overnight cultures of S.b.-mSA grown in YPD were centrifuged at 3000 rpm for 3 minutes and resuspended in PBS containing 0.05% Tween 20 (PBST). Cells were diluted in PBST to reach a final OD600 = 0.05. For each condition, 1 mL of diluted cells were centrifuged at 13,000 rpm for 1 minute. For detection of mSA expression on the cell surface, the cell pellet was resuspended in 50L of 1:50 dilutions of anti-HA-Alexa Fluor 488 and anti-FLAG-Alexa Fluor 594 in PBST. Cells were incubated with the antibodies for 30 minutes at room temperature, washed with PBST, then subject to flow cytometric analysis. For the biotinylated antibody dissociation constant (KD) measurements, cells pellets were prepared as described then resuspended in 50L of the indicated biotinylated antibodies at concentrations ranging from 1000 nM to 6 pM in PBST. A non-biotinylated anti-fibronectin antibody (Abcam, ab2413) was used as a control. Cells were incubated at room temperature for 30 minutes. Cells were then washed with PBST and resuspended in 50L of a 1:100 dilution of anti-rabbit-Alexa Fluor 594 for 30 minutes on ice. Cells were then washed with PBST and subject to flow cytometric analysis.

Instrument

LSRII Fortessa

Software

BD FACSDiva

Cell population abundance

Approximately 9,000 gated events were recorded for each marker in biological triplicates.

Gating strategy

Positive cells were identified by comparing with blank cell samples.

- [x] Tick this box to confirm that a figure exemplifying the gating strategy is provided in the Supplementary Information.
